# Optimizing antimicrobial susceptibility testing: cost and environmental benefits of MIC volume reduction

**DOI:** 10.1128/aac.00704-25

**Published:** 2025-10-17

**Authors:** Jonathan Clarhaut, Jeremy Moreau, Tom Collet, Emma Babiard, Vincent Aranzana-Climent, Sandrine Marchand, Kevin Brunet, Julien M. Buyck

**Affiliations:** 1 Université de Poitiers, PHAR2, Inserm U1070, Poitiers, France; 2 Laboratoire de Toxicologie et de Pharmacocinétique, CHU de Poitiers 36655 , Poitiers, France; 3 Service de Parasitologie et Mycologie Médicale, CHU de Poitiers 36655 , Poitiers, France; Providence Portland Medical Center, Portland, Oregon, USA

**Keywords:** MIC, miniaturization, small volume, Gram-negative, Gram-positive, yeast, antimicrobials

## Abstract

The determination of minimum inhibitory concentrations (MICs) is essential for evaluating antimicrobial efficacy, guiding both clinical treatment decisions and drug development. The standard broth microdilution method is widely used but requires significant reagent volumes, which can be limiting when working with novel or expensive antimicrobials. This study assesses the feasibility of reducing assay volumes without compromising MIC accuracy. We compared the MIC values obtained in standard 96-well plates (100 and 200 µL) to those in 384-well plates with reduced volumes (30 and 50 µL) for a range of ATCC gram-negative and gram-positive bacteria, as well as yeast species. Our results demonstrate that, except for micafungin against yeast, MIC values obtained with reduced volumes remained within the acceptable variability ranges defined by EUCAST and CLSI. The consistency of the MIC evaluation in 30 µL final volume was then confirmed against a selection of gram-negative 30 clinical isolates. Evaporation, a potential source of bias in smaller volumes, was mitigated by conducting experiments in a water-saturated atmosphere. Furthermore, reduced assay volumes significantly lowered material costs and antimicrobial consumption. This miniaturization approach provides a cost-effective and high-throughput alternative for antimicrobial susceptibility testing, ensuring accuracy and reproducibility and is particularly advantageous in research settings where compound availability is limited or associated with high costs.

## INTRODUCTION

The determination of minimal inhibitory concentration (MIC), which refers to the lowest concentration of an antimicrobial agent required to inhibit visible growth of a microorganism, plays a crucial role in the evaluation of antimicrobial efficacy, guiding both clinical treatment decisions and the development of novel therapeutics. Among the various methods for determining MIC, the broth microdilution technique has gained prominence due to its accuracy, reproducibility, and adaptability in a laboratory setting, and it is now generally considered the gold standard method by institutions such as the European Committee on Antimicrobial Susceptibility Testing (EUCAST) (1) or the Clinical and Laboratory Standards Institute (CLSI) (2) in the USA.

Generally performed in 96-well microtiter plates, the microdilution method enables the testing of multiple antibiotic or antifungal concentrations simultaneously, thereby providing a comprehensive antimicrobial profile for both established and novel compounds. This method is generally automated in clinical laboratories but not typically in microbiology research laboratories. Based on the recommendations of the CLSI (3, 4) or the EUCAST (1), this method is applied in a final volume of 100 µL to 200 µL in accordance with ISO 20776-1 (5).

Although wholly standardized in terms of the medium used, microorganism inoculum, incubation time, and temperature, the reproducibility and reliability of this method in smaller volumes—particularly in the context of testing new molecules or molecules synthesized in small amounts, or when using expensive broths like iron-depleted broth used to test the newly marketed cephalosporin siderophore cefiderocol—need to be validated. By employing smaller volumes, microdilution not only conserves resources (by reducing plastic consumption) but also enhances experimental efficiency, reducing costs and increasing throughput. This makes it especially beneficial for laboratories working with large panels of antimicrobials or conducting high-throughput screening for research in drug development.

In this article, we conducted a comparative study on MIC results when testing volumes were reduced. To do so, the values of MIC done in 200, 100, 50, and 30 µL final volume in two different microtiter plates (96 and 384 wells) were compared with those of the ATCC reference strains for five different bacterial species (*Pseudomonas aeruginosa*, *Staphylococcus aureus*, *Escherichia coli*, *Klebsiella pneumoniae*, and *Acinetobacter baumannii*), as well as two yeast species (*Candida krusei* and *Candida parapsilosis*), and with different antimicrobials (amikacin, gentamicin, ciprofloxacin, aztreonam, cefiderocol, ceftazidime, meropenem, linezolid, amphotericin B, fluconazole, and micafungin) that are representative of the main family used in clinics. The pronounced evaporation due to miniaturization of the final volume was evaluated and counteracted by incubation in a water-saturated environment. Consistency of MIC evaluation in small final volumes was then confirmed against a selection of 10 clinical isolates per gram-negative species.

## MATERIALS AND METHODS

### Strains used

The following reference strains were used for the study: *Pseudomonas aeruginosa* ATCC 27853, *Staphylococcus aureus* ATCC 29213, *Escherichia coli* ATCC 25922, *Klebsiella pneumoniae* ATCC 43816, *Acinetobacter baumannii* ATCC 19606*, Candida krusei* ATCC 6258, and *Candida parapsilosis* ATCC 22019. Ten clinical isolates with different resistance profiles were selected for *Pseudomonas aeruginosa*, *Escherichia coli*, and *Klebsiella pneumoniae* (Table S1).

### Antibiotics and reagents

Amikacin and gentamicin were provided from Acros (Thermo Fisher Scientific, Illkirch-Graffenstaden, France) and Carl Roth (Lauterbourg, France), respectively. Amphotericin B, aztreonam, ceftazidime, ciprofloxacin, fluconazole, linezolid, meropenem, and micafungin were purchased from Sigma-Aldrich (Saint-Quentin Fallavier, France). Cefiderocol was supplied as Fetroja by Shionogi.

Cation-adjusted Mueller Hinton Broth 2 (Ca-MHB) and RPMI 1640 (with L-glutamine, pH indicator, no bicarbonate supplemented with 2% [wt/vol] glucose and buffered to pH 7 with 0.165 mol/L MOPS) were purchased from Sigma-Aldrich.

Iron-depleted MHB (ID-MHB) was homemade from Ca-MHB according to the European Committee on Antimicrobial Susceptibility Testing (EUCAST) recommendations (6). The concentration of ferric ion was then measured by inductively coupled plasma mass spectrometry (ICP-MS) and was found to be 8.29 µg/L for this batch.

### Evaluation of evaporation

To ensure repeatability, all experiments were conducted using the Assist Plus robot and automated pipettes (Integra Biosciences SAS, Cergy Pontoise, France). Two types of plates were used: 96-well plates for tests with 100 and 200 µL final volume and 384-well plates for tests with 30, 50, and 100 µL. To evaluate the impact of evaporation, plates with different final volume of Ca-MHB were incubated for 24 h in air atmosphere (plates were deposited in incubator at 37°C without agitation) or in water-saturated atmosphere (plates were placed in a box with water reservoir and in the incubator at 37°C without agitation), without the addition of bacteria. Briefly, to measure the remaining volume after 24 h of incubation at 37 ± 2°C, either in an air or a water-saturated atmosphere, the content of each well was transferred into a 1.5 mL microtube and then weighed on a precision balance (Mettler Toledo, XPR6UD5, Viroflay, France). Each experiment has been replicated three times.

### MIC in microdilution

Minimum inhibitory concentrations (MICs) were determined following guidelines for standard conditions, with adjustments made as follows:

To assess the influence of the atmosphere, MIC was evaluated in 100 µL final volume in both air and water-saturated atmospheres for the five bacterial species, using different antibiotics as detailed in Table 1 (condition A).

**TABLE 1:** Conditions tested in MIC to evaluate the influence of the incubation atmospheres (A) and/or final volumes (B)

Antibiotics	Amikacin	Gentamicin	Ciprofloxacin	Ceftazidime	Meropenem	Aztreonam	Linezolid	Cefiderocol
*P. aeruginosa*	A, B	^*a*^ –	A, B	A, B	A, B	A, B	–	B
*K. pneumoniae*	A, B	–	A, B	–	A, B	–	–	B
*E. coli*	A, B	–	A, B	–	A, B	–	–	B
*A. baumannii*	A, B	–	A, B	–	A, B	–	–	–
*S. aureus*	–	A, B	A, B	–	A, B	–	A, B	–

^*a*^ –, non-tested condition.

To assess the influence of the smaller final volume, two types of plates were used: 96-well plates for tests with 100 and 200 µL final volume and 384-well plates for tests with 30, 50, and 100 µL in water-saturated atmosphere. For each condition, a minimum of three independent experiments was performed, each in triplicate. When available, MIC values were compared with the acceptable range of expected values for quality control strains, as defined in the EUCAST QC Tables 14 (7) and AFST QC v7.0 (8). For bacteria, all tests (conditions are detailed in Table 1) were performed in Ca-MHB (except cefiderocol, for which MIC measurements were determined in ID-MHB) with a final inoculum of 5 × 10^5^ CFU/mL, and the plates were analyzed after 16–20 h of incubation at 37°C as recommended by EUCAST. For yeasts, the MICs for three antifungals (amphotericin B, fluconazole, and micafungin) against both species were determined in supplemented RPMI 1640, using a final inoculum of 0.5–2.5 × 10^5^ CFU/mL. The plates were analyzed after 24 h of incubation at 37°C as recommended by EUCAST (6). A double reading—visual inspection and optical density measurement using a plate reader (Tecan Infinite M200, Männedorf, Switzerland)—was performed to minimize difficulties in endpoint determination.

## RESULTS

### Impact of the incubation atmosphere on evaporation of the medium

**Fig 1:**
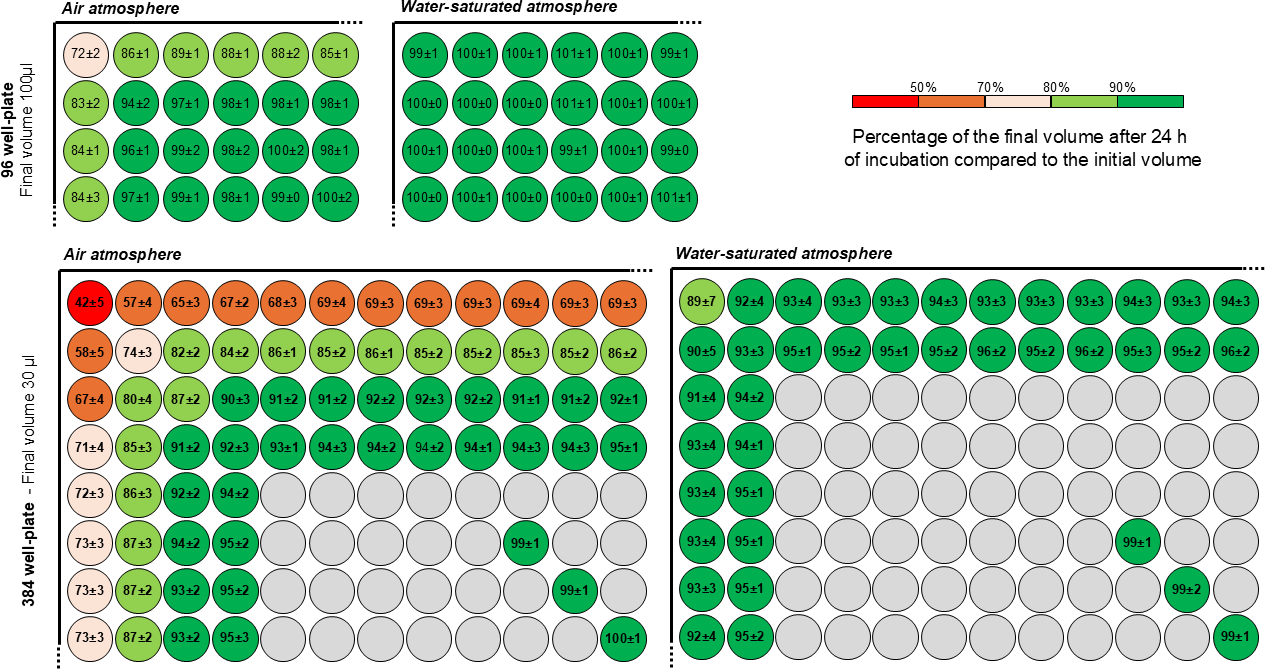
Percentage of evaporation at 24 h in air and water-saturated atmospheres. 96-well plate with final volume of 100 µL and 384-well plate with final volume of 30 µL were incubated for 24 h at 37°C without agitation. Results are expressed in percentage of final volume at 24 h compared to initial volume. The figure shows a schematic representation of the top left-hand corner of a microtiter plate.

To compare evaporation, volumes were measured for all peripheral wells and for the wells in the middle of the plate. Figure 1 shows that in an air atmosphere, 72 ± 2% of the volume remained in the corner wells, and that after 24 h of incubation in 96-well plates, more than 90% of the volume remained in the edge wells. No volume loss was observed for the middle wells in the plate. For all wells, the final volumes remained stable in a water-saturated atmosphere (100% of the initial volume was found at 24 h).

For the 384-well plates with a final volume of 30 µL, the effect was more pronounced, with only 42 ± 5% of the final volume remaining in the corner wells after 24 h in an air atmosphere. For the edge wells (first column and first row), the final volume ranged from 57 ± 4% to 73 ± 3% at 24 h, whereas the final volumes were close to 100% in the third column and third row, extending to the middle wells in the plate. For all wells in 384-well plates, the final volume remained above 90% in a water-saturated atmosphere. The results for all conditions tested (96-well versus 384-well plates and different volumes) are presented in Fig. S1. The results were similar for the other areas of the corresponding plates. To limit the evaporation phenomenon, it was decided to maintain a water-saturated atmosphere for the remainder of the study.

### Impact of the incubation atmospheres on MIC values in 100 µL final volume

**Fig 2:**
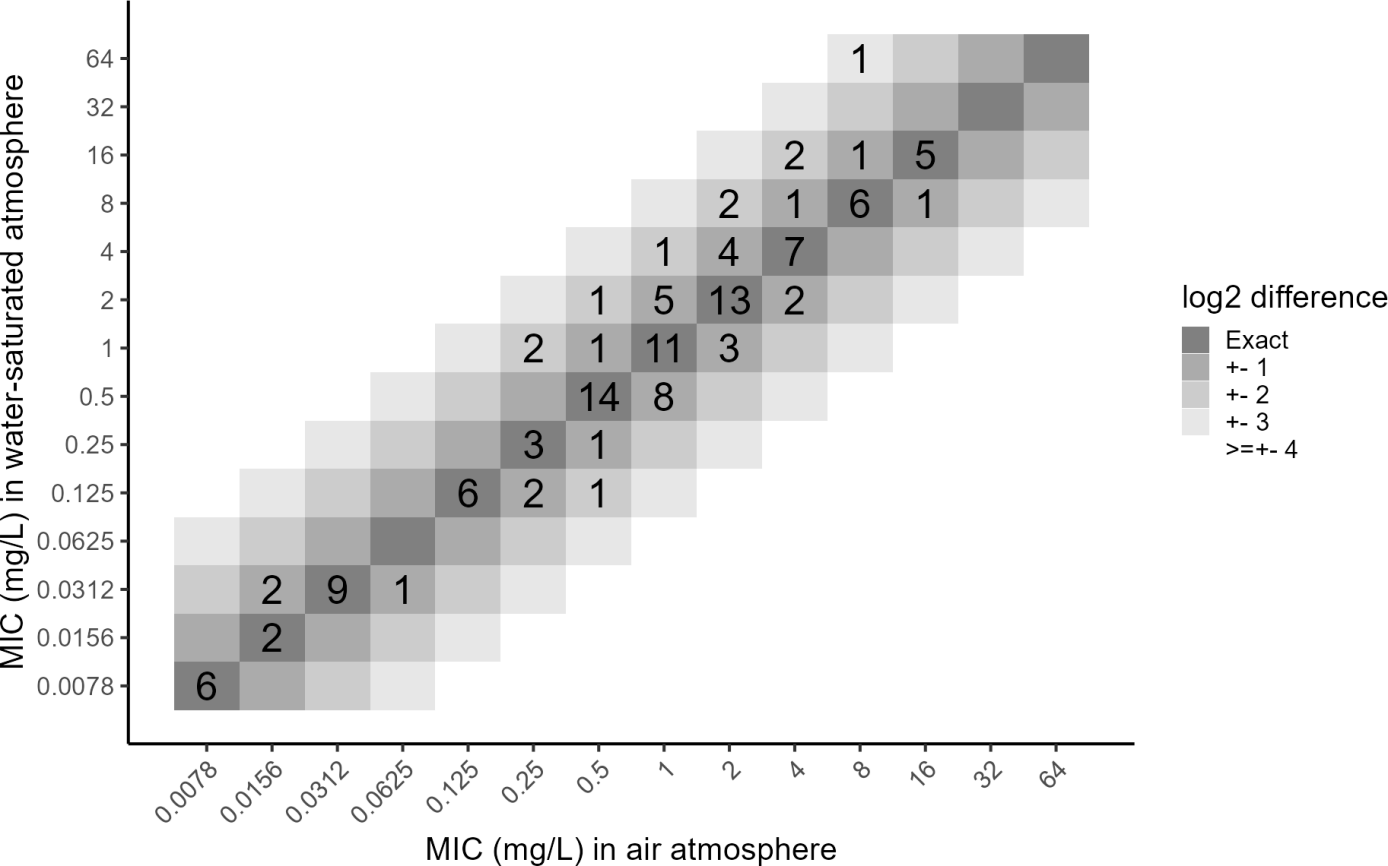
Comparison of MIC values obtained in 100 µL final volume in air or water-saturated atmosphere. MIC measurements were paired for both atmospheres and repeated in three independent experiments. The different gray levels show differences of log_2_ dilution from 0 (exact) to ≥ ±4 log_2_ dilutions.

To ensure that a water-saturated atmosphere did not influence MIC measurement, MIC values obtained in a final volume of 100 µL in both air and in a water-saturated atmospheres were compared. As illustrated in Fig. 2, 91.9% (114 out of 124 measurements) of the MIC values fell within the acceptable ±1 log₂ dilution range, while only 10 values (8.1%) were over the ±2 log₂ dilution range.

### Impact of the volume reduction on MIC values

**Fig 3:**
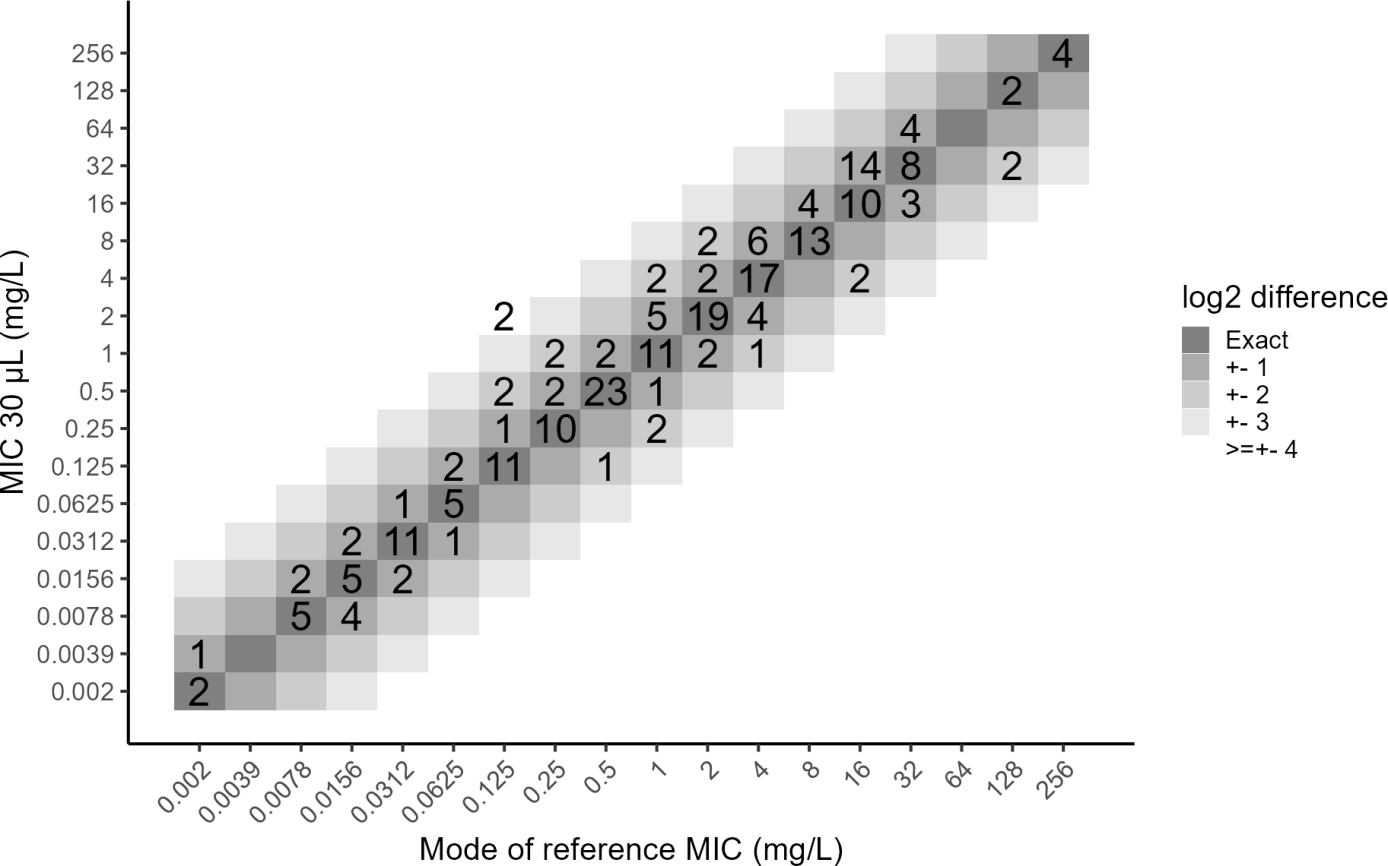
Comparison of MIC values obtained in standard final volume (reference MIC) and in 30 µL final volume. MIC values of all antimicrobials against all bacterial and fungal species were plotted in differences of MIC values at 30 µL against the mode of MIC values obtained with standard (100/200 µL) final volumes. The different gray levels show differences of log_2_ dilution from 0 (exact) to ≥ ±4 log_2_ dilutions.

**Fig 4:**
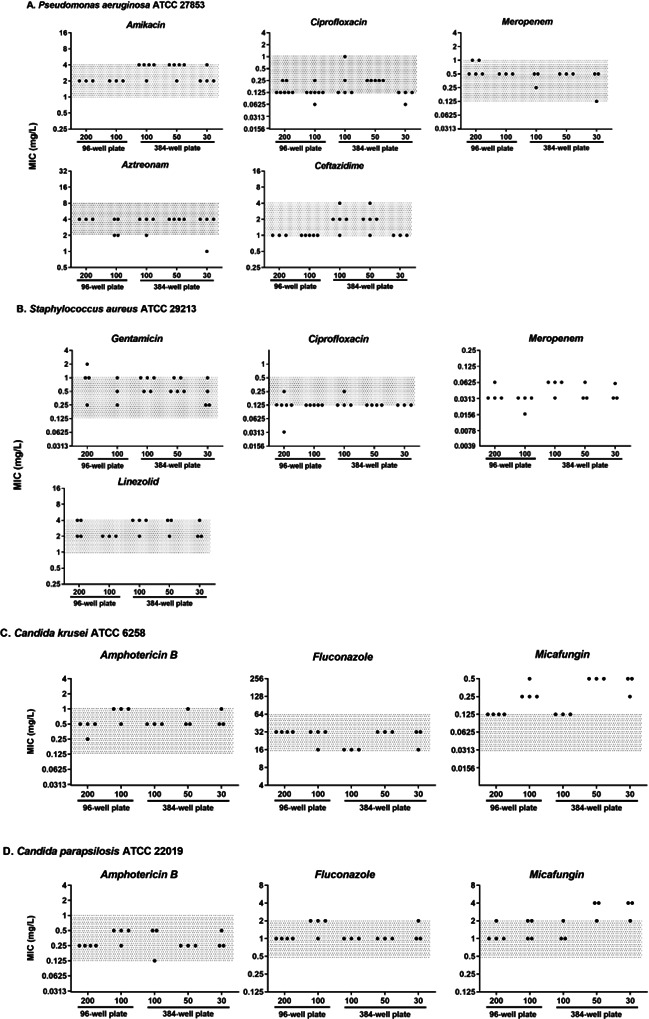
Comparison of MIC values depending on the different conditions. (**A**) MIC values of amikacin, ciprofloxacin, meropenem, aztreonam, and ceftazidime against *Pseudomonas aeruginosa* ATCC 27853. (**B**) MIC values of ciprofloxacin, gentamicin, linezolid, and meropenem against *Staphylococcus aureus* ATCC 29213. (**C**) MIC values of amphotericin B, fluconazole, and micafungin against *Candida krusei* and (**D**) *Candida parapsilosis*. Each MIC was evaluated at least three independent times (black dot). The gray area represents the acceptable range for the respective quality control strains according to the EUCAST.

MIC measurements were then assessed in different volumes with different well plates for four gram-negative species (*Pseudomonas aeruginosa*, *Klebsiella pneumoniae*, *Escherichia coli*, and *Acinetobacter baumannii*), one gram-positive (*Staphylococcus aureus*), and two Candida species (*Candida krusei* and *Candida parapsilosis*) for different antimicrobials representative of the different families used in therapy. Results are presented in Fig. 3 and 4; Fig. S2.

Figure 3 compares the MIC values obtained in 30 µL final volumes with the MIC values obtained in standard final volume, regardless of the species and antimicrobial tested. Most MIC values (150/222, 66.4%) showed an exact match between the MIC values obtained in both final volumes, while 54/222 (24.3%) showed a ± 1 log₂ dilution difference, representing 90.7% of the values that fell in the acceptable range of MIC. However, 16/222 (7.2%) values showed a ± 2 log₂ dilution difference. Two MIC value differences greater than ±2 log₂ dilutions were observed.

For *Pseudomonas aeruginosa* (Fig. 4A), no significant difference was observed in MIC values, regardless of the volume used for the measurement. All MIC values were within the acceptable range (gray area) for the quality control strain, as defined by EUCAST. Comparable MIC values were obtained for *Staphylococcus aureus*, irrespective of the volume used for MIC evaluation (Fig. 4B). For *Candida* sp. (Fig. 4C and D), results were highly comparable, with stable MIC values regardless of the final volume for amphotericin B and fluconazole. However, MIC values tended to be slightly increased by one to two dilutions for smaller volumes (30 and 50 µL) in the 384-well plates, with three out of three measurements for *C. krusei* and two out of three measurements for *C. parapsilosis* showing an increase for micafungin compared to the 200 µL volume in 96-well plates. The results for other gram-negative species (*Escherichia coli* ATCC 25922, *Acinetobacter baumannii* ATCC 19606, and *Klebsiella pneumoniae* ATCC 43816) on a restricted selection of antibiotics are presented in Fig. S2 and showed no significant impact of the final volume on the MIC values of three different antibiotics: amikacin, ciprofloxacin, and meropenem.

### Validation on clinical isolates

**Fig 5:**
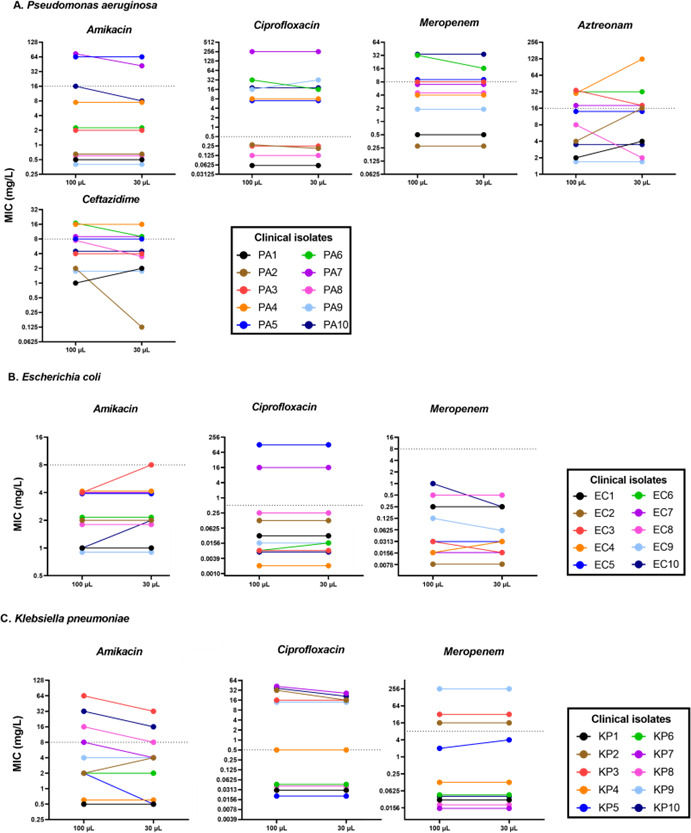
Influence of final volume on the measurement of MIC values for clinical isolates. (**A**) MIC values of amikacin, ciprofloxacin, meropenem, aztreonam, and ceftazidime against 10 *Pseudomonas aeruginosa* clinical isolates. (**B**) MIC values of amikacin, ciprofloxacin, and meropenem against 10 *Escherichia coli* clinical isolates. (**C**) MIC values of amikacin, ciprofloxacin, and meropenem against 10 *Klebsiella pneumoniae* clinical isolates. The dotted lines represent the “R” limit of clinical breakpoint for the respective bacterial species and antibiotics according to the EUCAST.

MICs were determined in both standard and 30 µL final volumes for 10 *Pseudomonas aeruginosa*, 10 *Klebsiella pneumoniae*, and 10 *Escherichia coli* clinical isolates, each displaying different resistance profiles (Table S1). Representative therapeutic antibiotics were tested, and results are shown in Fig. 5. Overall, while some variations in MIC values were observed between the two volumes, 93.6% (103/110) of the measurements remained within ±1 log₂ dilution. Importantly, no isolate–antibiotic combination showed a categorical shift from resistant to susceptible or vice versa.

### Reducing volume is associated with cost savings

As reducing the volume for MIC measurement could have a major impact in terms of cost and the quantity of antimicrobial used, cefiderocol, for which expensive media (ID-MHB) is used, was also evaluated without showing any difference in MIC (Fig. S3). On the other hand, a cost saving of around 15% was estimated when the volume was reduced for MIC evaluation of common antimicrobials, and it can exceed 25% when expensive drugs such as cefiderocol are used (Tables S2 and S3). This miniaturization also has an important environmental impact, as it reduces the amount of plastic required to perform this experiment by 69.3% (Table S4).

## DISCUSSION

Optimizing MIC measurement conditions by reducing the final assay volume is a key challenge for future research. This approach aims to enable the testing of a larger number of strains while minimizing costs associated with novel molecules, specialized media (which can be highly expensive), or synthesized compounds available only in limited quantities. In this study, we demonstrated that reducing the final assay volume to 30 µL generally does not significantly impact MIC values across various antimicrobials tested against gram-negative and gram-positive bacteria, as well as yeast species.

### The evaporation is more pronounced with small volume but can be counteracted in a water-saturated atmosphere

A potential bias introduced by reducing the final assay volume is evaporation. In this study, we evaluated evaporation and found it to be more pronounced at smaller volumes (30 µL in 384-well plates), with the effect being most significant in corner and edge wells but rapidly becoming negligible in other wells. To mitigate evaporation in edge wells, we compared evaporation under identical conditions in microwell plates incubated in a water-saturated atmosphere, which significantly reduced the effect. Moreover, the MIC values of antibiotics measured in both air and water-saturated atmospheres were not significantly different (Fig. 2). Therefore, to minimize evaporation at low final volumes, experiments should be conducted in a water-saturated atmosphere. An alternative to prevent evaporation would be to use sterile sealing films; however, this approach would increase experimental costs.

### Reducing the volume of experiments does not significantly influence MIC values

MIC measurements were then performed using standard volume in 96-well plates versus reduced volume in 384-well plates on a broad selection of bacterial and fungal species, as well as a wide range of antimicrobials. MIC values obtained under these different conditions were compared to the standard condition (96-well plates, 100 or 200 µL final volume) and were within the acceptable value range of the corresponding quality control strains as defined by EUCAST, when available (*P. aeruginosa*, *E. coli*, *S. aureus*, *C. parapsilosis*, and *C. krusei*). Most MIC values fell within the acceptable ±1 log₂ dilution range, indicating that the final volume and plate format did not significantly influence the measurement.

In this study, all plates were freshly prepared and used immediately without freezing. Although the potential impact of plate freezing was not investigated in this study, it could influence assay consistency in other settings and may warrant further evaluation.

The MICs for micafungin were at or above the acceptable limit. Micafungin is a sticky agent that tends to bind to plastics. A recent study (9) found that the number of tip changes during testing can influence the MIC values, probably due to the drug’s binding to plastic surfaces. In another study, the authors showed that the type of polystyrene used in microdilution plates could influence the MIC of caspofungin (10). These interactions could explain the discrepancies observed in our study. Since smaller volumes of micafungin were used in smaller wells, a greater proportion of the drug molecules may bind to the plastic, leading to a more significant impact on the MIC. This phenomenon is particularly pronounced when the MIC is low, as seen with a higher impact on *C. krusei* than on *C. parapsilosis*. These results suggest that, for accurate comparisons of echinocandin MICs, laboratories should follow a consistent protocol, and that the use of lower volumes may not be advisable.

### To reduce the volume of experiments and reduce materials needed and cost of the experiments

We then estimated the savings resulting from reducing the volume of experiments, from 100 µL in 96-well plates to 30 µL in 384-well plates.

Although the savings for a series of MIC measurements with “standard” antibiotics (estimated for two strains against five antibiotics) are relatively modest, with a 15.1% cost reduction for 384-well plates with a 30 µL final volume (Table S2), they are considerably higher when expensive drugs requiring special media are used. In our example, for 10 bacterial strains with cefiderocol, cost savings could exceed 25.1% for 384-well plates with a 30 µL final volume (Table S3). It should be noted that the cost estimates in this study were calculated using the pharmaceutical formulation of cefiderocol (Fetroja), which is priced at approximately €1500 per gram in Europe. Importantly, the potential cost savings may be even greater when considering research-grade powders obtained from chemical suppliers (e.g., Sigma/Merck, MedChem Express), where prices can reach up to €100,000 per gram. These prices reflect the cost of high-purity compounds intended for experimental use rather than for clinical application.

Laboratory plastic consumption was estimated for an experiment on 100 strains, showing a usage of over 21.8 kg under standard conditions, versus 6.7 kg under reduced-volume conditions—corresponding to a 69.3% reduction in plastic waste (Table S4).

To our knowledge, this study is among the first to validate small-volume MIC testing based on the standards close to those recommended by EUCAST or CLSI. While other studies have proposed miniaturizing MIC testing to increase experimental throughput, these approaches often rely on novel methodologies that require specialized (and potentially expensive) equipment. For instance, Needs et al. recently demonstrated good agreement with CLSI standards using a miniaturized broth microdilution antibiotic susceptibility testing (AST) method based on microcapillary devices for gram-negative bacteria (11). Similarly, Choi et al. developed a rapid AST method using a single-cell approach combined with time-lapse microscopy imaging (12). However, in addition to their reliance on specialized equipment, these methods have limitations, particularly in their estimation of antimicrobial effects using a small number of bacteria. This may have overlooked the inoculum effect, which can significantly influence MIC values (13). Previously, a study proposed an approach similar to ours for miniaturizing checkerboards, but tested only a single bacterial species on beta-lactam/beta-lactamase inhibitor combinations (14).

### Potential limitations of reduced-volume MIC testing

Reducing assay volumes can improve efficiency and reduce reagent use but may introduce limitations. Very low volumes (e.g., 20 µL) reduce reproducibility due to pipetting accuracy constraints, as observed in our study (data not shown), where MIC values were less consistent at this volume. While further volume reduction cannot be excluded, the limit of reliable pipetting was reached with the pipettes used. Moreover, handling small volumes in 384-well formats typically requires automated pipetting systems and sensitive plate readers, which may not be accessible to all laboratories. Although manual pipetting remains feasible for 384-well plates, the use of pipetting robots appears essential to improve reproducibility, accuracy, and throughput. Additionally, endpoint determination for certain antibiotics and antifungals can be more challenging under reduced-volume conditions. A double reading—visual inspection and optical density measurement using a plate reader—helps minimize difficulties in endpoint determination. These limitations should be considered when applying reduced-volume MIC protocols.

Overall, our study showed that reducing the final volume does not affect MIC values (except for micafungin) for a wide range of bacterial and yeast species across various antimicrobials with different mechanisms of action. On the other hand, reducing test volume can generate consequent savings when assessing the activity of expensive molecules. Reducing the volume of MIC measurements would also make it possible to reduce the quantity of molecules needed, for example, in preliminary activity tests for new molecules that are difficult to synthesize or cannot be synthesized in large quantities.

## SUPPLEMENTAL MATERIAL

Supplemental material aac.00704-25-s0001.docx Fig. S1 to S3; Tables S1 to S4.

ASM does not own the copyrights to Supplemental Material that may be linked to, or accessed through, an article. The authors have granted ASM a non-exclusive, world-wide license to publish the Supplemental Material files. Please contact the corresponding author directly for reuse.
