# Supplementary material for: Optimizing antimicrobial susceptibility testing: cost and environmental benefits of MIC volume reduction
Source: Antimicrob Agents Chemother. 2025 Oct 17;69(12):e00704-25. doi: 10.1128/aac.00704-25 (PMC12691650; doi:10.1128/aac.00704-25)
Supplement: Supplemental material — Fig. S1 to S3; Tables S1 to S4. [file aac.00704-25-s0001.docx]

**Supplemental figures**

| **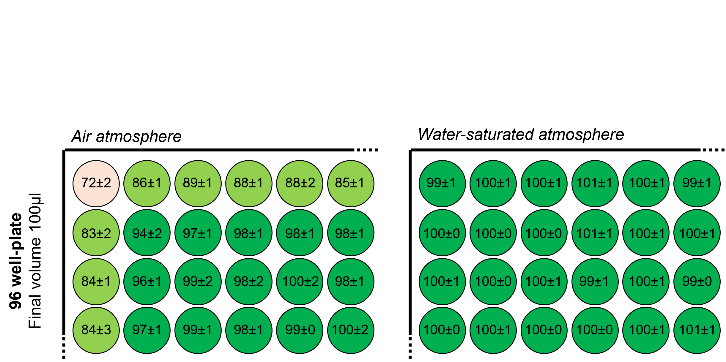** |
| --- |
| **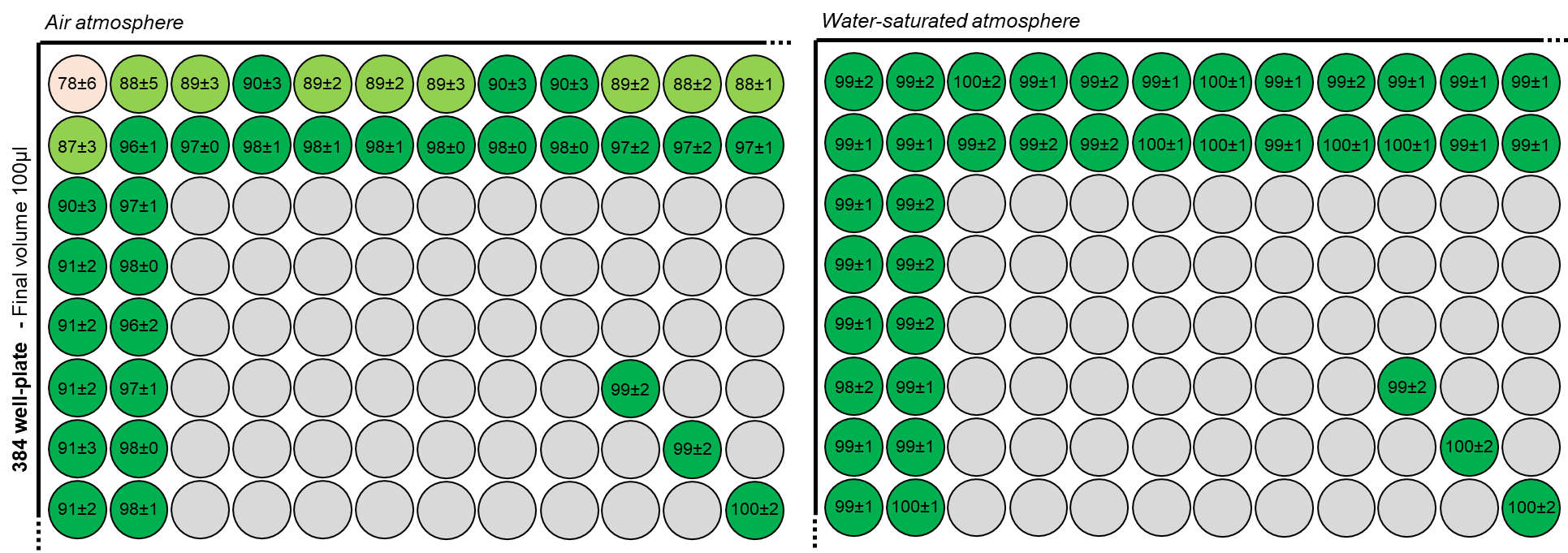** |
| **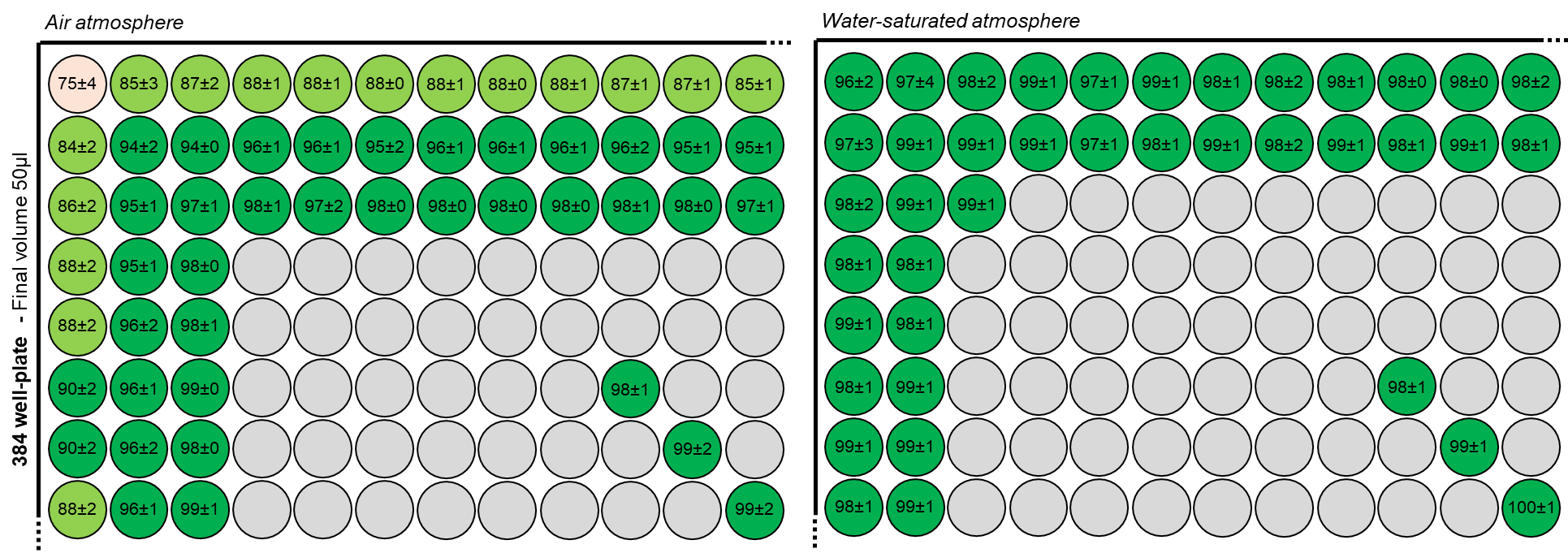** |
| Percentage of final volume after 24 h of incubation compared to the initial volume  90%  50%  80%  70% |

**Figure S1. Percentage of evaporation at 24 h in air** **and water-saturated atmospheres**. 96-well plate with final volume of 200 µL and 384-plate with final volume of 50 and 100 µL were incubated for 24 h at 37°C without agitation. Results are expressed in percentage of final volume at 24 h compared to initial volume. The figure shows a schematic representation of the top left-hand corner of a microtiter plate.

**
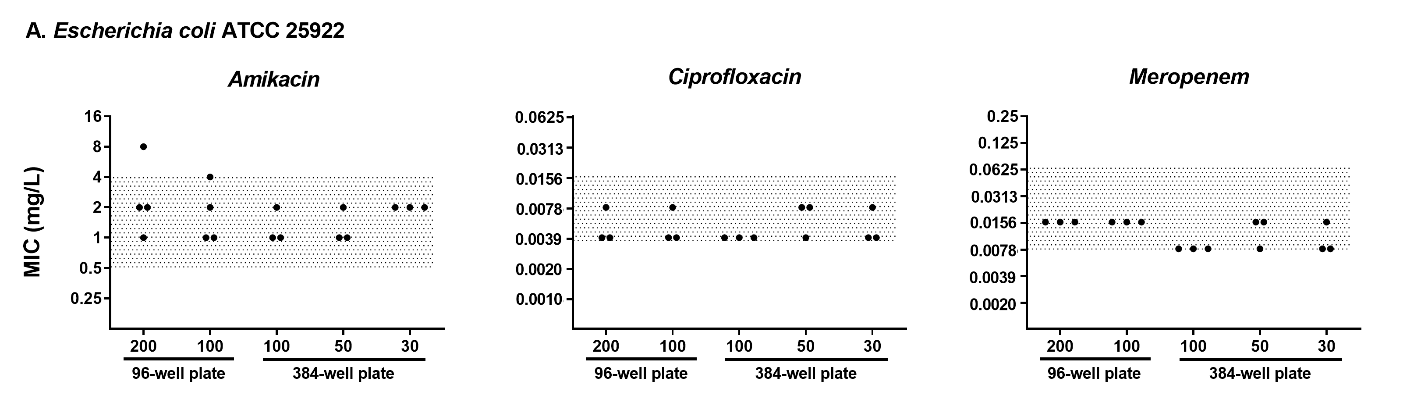

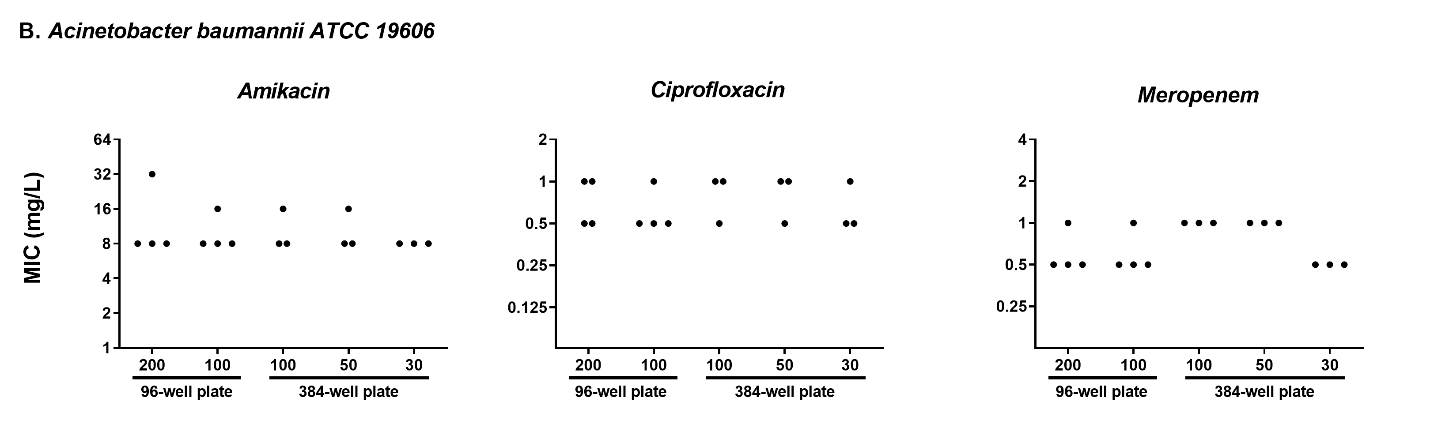

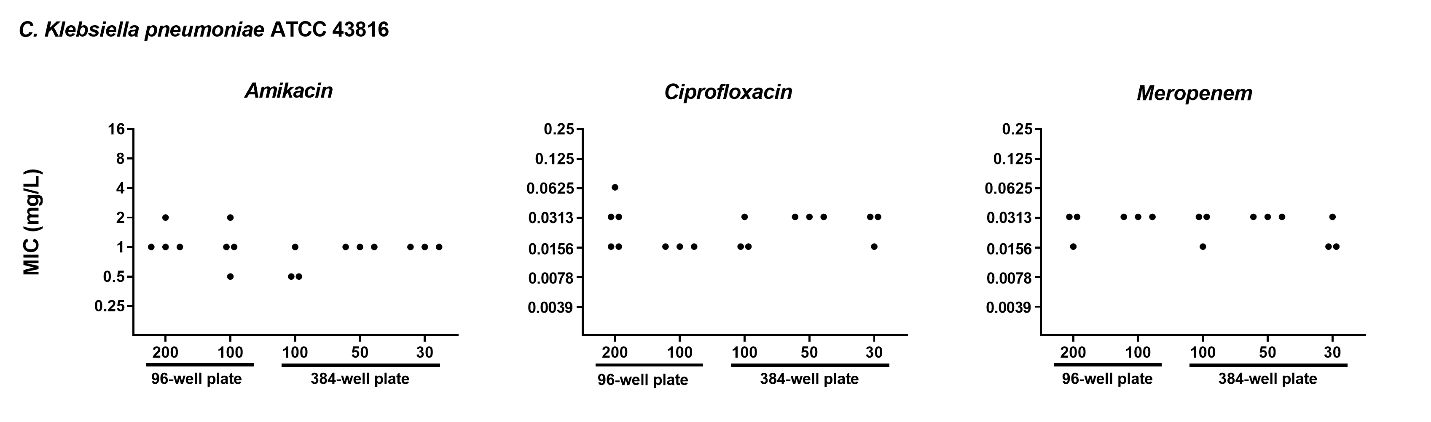
**

**Figure S2. Comparison of MIC values for amikacin, ciprofloxacin and meropenem depending on the different conditions. A.** MIC values against *Escherichia coli* ATCC 25922. **B.** MIC values against *Acinetobacter baumannii* ATCC 19606. **C.** MIC values against *Klebsiella pneumoniae* ATCC 43816. Each MIC has been evaluated at least three independent times (black dot). The grey area represents the acceptable range for the respective quality control strains according to the EUCAST when available.

**
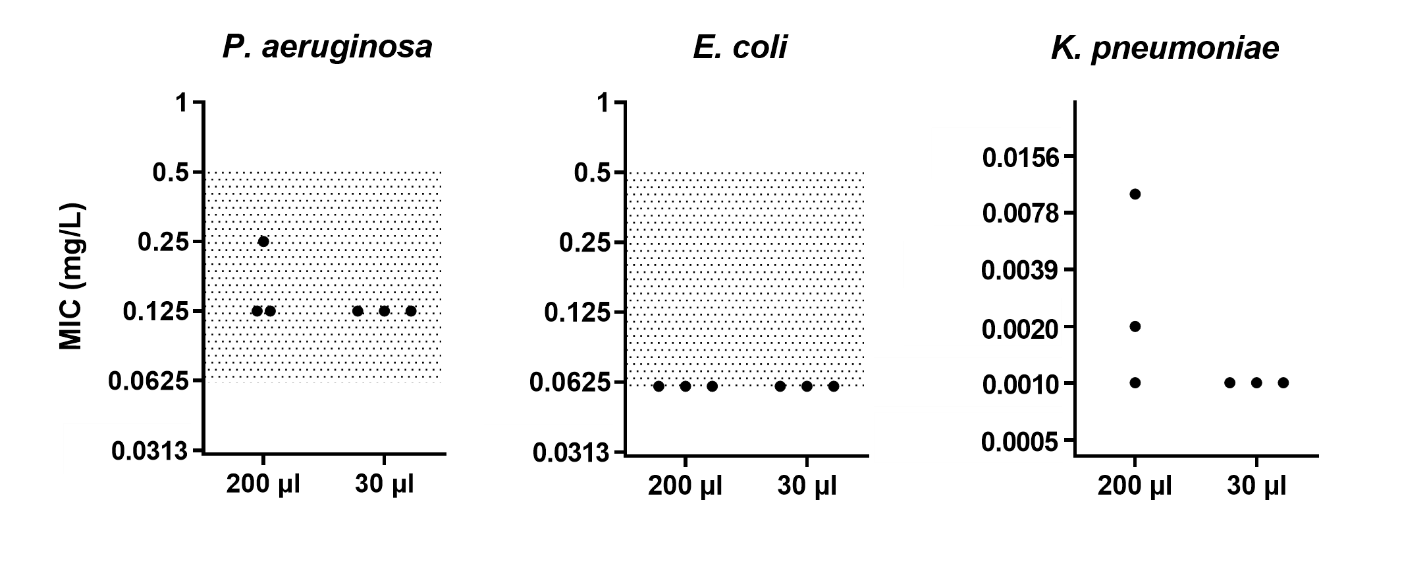
**

**Figure S3. Comparison of MIC values for cefiderocol in water-saturated atmosphere depending on the different conditions against *Pseudomonas aeruginosa*, *Escherichia coli* and *Klebsiella pneumoniae*.** Each MIC has been evaluated at least three independent times (black dot). The grey area represents the acceptable range for the respective quality control strains according to the EUCAST, when available.

| **Table S1. Caracteristics of clinical isolates used in the study.** | | |
| --- | --- | --- |
| **Species** | **Origin (Year of isolation)** | **Resistance Profile** |
| *Pseudomonas aeruginosa* | | |
| PA1 | Human/Blood (2019) | AMK: S ; CIP: S ; MER: S ; ATM: S ; CAZ: S |
| PA2 | Human/Blood (1999) | AMK: S ; CIP: S ; MER: S ; ATM: S ; CAZ: S |
| PA3 | Human (2001) | AMK: S ; CIP: S ; MER: S ; ATM: S ; CAZ: S |
| PA4 | Human (2001) | AMK: S ; CIP: S ; MER: S ; ATM: S ; CAZ: S |
| PA5 | Human/Blood (2015) | AMK : R ; CIP: R ; MER: R ; ATM: S ; CAZ: R |
| PA6 | Human (2001) | AMK : S ; CIP: R ; MER: R ; ATM: R ; CAZ: R |
| PA7 | Human (2015) | AMK : R ; CIP: R ; MER: R ; ATM: R ; CAZ: R |
| PA8 | Human/Lung (2015) | AMK : S ; CIP: S ; MER: S ; ATM: S ; CAZ: S |
| PA9 | Human/rectum (2018) | AMK : S ; CIP: R ; MER: S ; ATM: S ; CAZ: S |
| PA10 | Human/Urine (2020) | AMK : R ; CIP: R ; MER: R ; ATM: S ; CAZ: S |
|  |  |  |
| *Klebsiella pneumoniae* | | |
| KP1 | Human | AMK: S ; CIP: S ; MER: S |
| KP2 | Human | AMK: S ; CIP: R ; MER: S |
| KP3 | Human | AMK: R ; CIP: R ; MER: S |
| KP4 | Human | AMK: S ; CIP: R ; MER: S |
| KP5 | Human | AMK: S ; CIP: S ; MER: S |
| KP6 | Animal | AMK: S ; CIP: S ; MER: S |
| KP7 | Animal | AMK: S ; CIP: R ; MER: S |
| KP8 | Animal | AMK: S ; CIP: S ; MER: S |
| KP9 | Human/Blood (2021) | AMK: S ; CIP: R ; MER: R |
| KP10 | Human/Blood (2021) | AMK: R ; CIP: R ; MER: S |
|  |  |  |
| *Escherichia coli* | | |
| EC1 | - | AMK: S ; CIP: S ; MER: S |
| EC2 | - | AMK: S ; CIP: S ; MER: S |
| EC3 | Animal (2019) | AMK: S ; CIP: S ; MER: S |
| EC4 | - | AMK: S ; CIP: S ; MER: S |
| EC5 | - | AMK: S ; CIP: R ; MER: S |
| EC6 | Animal (2019) | AMK: S ; CIP: S ; MER: S |
| EC7 | Animal (2018) | AMK: S ; CIP: R ; MER: S |
| EC8 | Human | AMK: S ; CIP: S ; MER: S |
| EC9 | Human | AMK: S ; CIP: S ; MER: S |
| EC10 | Animal | AMK: S ; CIP: S ; MER: S |

**Table S2.** Estimation of the experimental cost for MIC measurements with 5 standard antibiotics against two bacterial strains.

| **Consumables** (calculated for 2 strains with 5 antibiotics in triplicate) | **96 well-plate  Final volume 100 µL** | **384 well-plate  Final volume 30 µL** |
| --- | --- | --- |
| Plates | 5 | 1 |
| Dilution plate | 1 | 1 |
| Plates | 4 € (5 + 1 plates) | 8 € (1 + 1 plate) |
| Medium - MHB | 0,39 € | 0,20 € |
| Tips | 10,75 € | 14 € |
| Antibiotics :  ATM (590 €/g) | 1,78 € | 0,30 € |
| MEM (600 €/g) | 3,60 € | 0,60 € |
| AMK (18 €/g) | 0,11 € | 0,018 € |
| CIP (7,8 €/g) | 0,03 € | 0,004 € |
| CAZ (62 €/g) | 0,38 € | 0,06 € |
| **For one experiment** | 27.3 € | 23.2 € |
|  | 15.1% savings | |

**Table S3.** Estimation of the experimental cost for MIC measurements with cefiderocol antibiotic against ten bacterial strains.

| **Consumables** (calculated for 10 strains with 1 antibiotic in triplicate) | **96 well-plate  Final volume 100 µL** | **384 well-plate  Final volume 30 µL** |
| --- | --- | --- |
| Plates | 5 | 1 |
| Dilution plate | 1 | 1 |
| Plates | 4 € (5 + 1 plates) | 8 € (1 + 1 plate) |
| Medium – ID-MHB (210 €/L) | 21 € | 7 € |
| Tips | 10,75 € | 14 € |
| Antibiotics :  Fetroja / CFD (1500 €/g) | 3.5 € | 0.4 € |
| **For one experiment** | 39.25 € | 29.4 € |
|  | 25.1% savings | |

**Table S4.** **Estimation of Plasticware Consumption for MIC Measurements on 100 Strains.**

| **Consumables** | **96 well-plate  Final volume 100 µL** | **384 well-plate  Final volume 30 µL** |
| --- | --- | --- |
| Plates | 15500 g (250 * 62 g) | 4000 g (50 * 80 g) |
| Dilution plate | 620 g (10 * 62 g) | 620 g (10 * 62 g) |
| Tips for antibiotic and inoculum preparation | 425 g (1700 * 0.25 g) | 425 g (1700 * 0.25 g) |
| Tips for dilution and spreading of bacterial inocula | 275 g (1100 * 0.25 g) | 275 g (1100 * 0.25 g) |
| Tips for plate preparation | 112 g (400 * 0.28 g)  4875 g (19500 * 0.25 g) | 1008 g (3600 * 0.28 g)  375 g (1500 * 0.25 g) |
|  |  |  |
| **Total** | 21807 g | 6703 g |
|  | 69.3 % savings | |

The weight of different plasticwares have been estimated as follows: 96-well plate = 62 g; 384-well plate = 80 g, 200 µL pipette tips = 0.25 g; 200 µL pipette tips for robot = 0.28 g
